# Supplementary material for: The Effect of the 14:10-Hour Time-Restricted Feeding (TRF) Regimen on Selected Markers of Glucose Homeostasis in Diet-Induced Prediabetic Male Sprague Dawley Rats
Source: Nutrients. 2025 Jan 15;17(2):292. doi: 10.3390/nu17020292 (PMC11768421; doi:10.3390/nu17020292)
Supplement: Supplementary file 1 [file nutrients-17-00292-s001.zip › nutrients-3360025-supplementary.pdf]

Supplementary Materials: DIET COMPOSITIONS

Table S1: Composition of the high fats high carbohydrates (HFHC) diet

Ingredient	Incl(%)	Mix(kg)
Maize	38.98	390.000
Palm Oil	20.99	210.000
Soya Full Fat	14.99	150.000
Wheat Gluten	6.50	65.000
Flour	6.00	60.000
Monodex	5.00	50.000
Sugar - White	5.00	50.000
Limestone	1.00	10.000
Dicalcium Phosphate	0.50	5.000
Vitamin Premix	0.35	3.500
Salt - Fine	0.30	3.000
Amino Acid - DL Methionine	0.30	3.000
Mineral Premix	0.10	1.000
	**100.01**	**1000.50**

Table S2: Nutritional value of the high fats high carbohydrates (HFHC) diet

Nutrient	Units	Actual
Dry Matter	g/kg	919.93
Metabolizable Energy	MJ/kg	15.86
Crude Protein	g/kg	151.27
AShreonine	g/kg	4.51
ASIsoluecine	g/kg	5.24
ASLysine	g/kg	6.54
ASMethionine	g/kg	4.86
ASryptophan	g/kg	1.30
ASstidine	g/kg	3.30
ASTSAA	g/kg	6.79
ASValine	g/kg	5.80
Fat	g/kg	250.46
Carbohydrate	g/kg	427.29
Fibre	g/kg	22.08
Ash	g/kg	26.31
Avl Phosphorus	g/kg	1.66
Calcium	g/kg	5.47
Total Phosphorus	g/kg	3.60

Table S3: The overall caloric contribution from fats, proteins, and carbohydrates in a high-fat high-carbohydrate diet.

Carbohydrates	219.92 kcal/g
Fats	323.82 kcal/g
Proteins	27.2 kcal/g
Total	570.94 kcal/g

Table S4: The overall caloric contribution from fats, proteins, and carbohydrates in a standard diet.

Carbohydrates	155.92 kcal/g
Fats	134.91 kcal/g
Proteins	27.2 kcal/g
Total	318.03 kcal/g

Table S5: Composition of fats, proteins, and carbohydrates of the standard diet.

Fats	15%
Proteins	30%
Carbohydrates	35%
Other components	20%
